# Morphologic and Gene Expression Criteria for Identifying Human Induced Pluripotent Stem Cells

**DOI:** 10.1371/journal.pone.0048677

**Published:** 2012-12-13

**Authors:** Shohei Wakao, Masaaki Kitada, Yasumasa Kuroda, Fumitaka Ogura, Toru Murakami, Akira Niwa, Mari Dezawa

**Affiliations:** 1 Department of Stem Cell Biology and Histology, Tohoku University Graduate School of Medicine, Sendai, Miyagi, Japan; 2 Department of Anatomy and Anthropology, Tohoku University Graduate School of Medicine, Sendai, Miyagi, Japan; 3 Center for iPS Cell Research and Application, Kyoto University, Kyoto, Japan; University of Melbourne, Australia

## Abstract

Induced pluripotent stem (iPS) cells can be generated from somatic cells by the forced expression of four factors, Oct3/4, Sox2, Klf4, and c-Myc. While a great variety of colonies grow during induction, only a few of them develop into iPS cells. Researchers currently use visual observation to identify iPS cells and select colonies resembling embryonic stem (ES) cells, and there are no established objective criteria. Therefore, we exhaustively analyzed the morphology and gene expression of all the colonies generated from human fibroblasts after transfection with four retroviral vectors encoding individual factors (192 and 203 colonies in two experiments) and with a single polycistronic retroviral vector encoding all four factors (199 and 192 colonies in two experiments). Here we demonstrate that the morphologic features of emerged colonies can be categorized based on six parameters, and all generated colonies that could be passaged were classified into seven subtypes in colonies transfected with four retroviral vectors and six subtypes with a single polycistronic retroviral vector, both including iPS cell colonies. The essential qualifications for iPS cells were: cells with a single nucleolus; nucleus to nucleolus (N/Nls) ratio ∼2.19: cell size ∼43.5 µm^2^: a nucleus to cytoplasm (N/C) ratio ∼0.87: cell density in a colony ∼5900 cells/mm^2^: and number of cell layer single. Most importantly, gene expression analysis revealed for the first time that endogenous Sox2 and Cdx2 were expressed specifically in iPS cells, whereas Oct3/4 and Nanog, popularly used markers for identifying iPS cells, are expressed in colonies other than iPS cells, suggesting that Sox2 and Cdx2 are reliable markers for identifying iPS cells. Our findings indicate that morphologic parameters and the expression of endogenous Sox2 and Cdx2 can be used to accurately identify iPS cells.

## Introduction

Embryonic stem (ES) cells derived from the inner cell mass of blastocysts are able to self-renew and differentiate into cells representative of all three germ layers, indicating that they are pluripotent stem cells [1], [2]. While they are expected to contribute to cell-based therapy due to their ability to differentiate into a great variety of cells, ethical considerations relating to the use of fertilized eggs pose limitations for their practical use. Induced pluripotent stem (iPS) cells can be generated from adult human somatic cells by introducing factors such as Oct3/4, Sox2, Klf-4, and c-Myc (the four so-called Yamanaka factors), and like ES cells, iPS cells are able to self-renew and differentiate into cells representative of all three germ layers [3]. iPS cells have many advantages and the ethical concerns regarding the use of fertilized eggs are eliminated. Disease-specific iPS cells generated from patients are also expected to be applicable for the evaluation of disease mechanisms and drug efficacy [4], [5].

Introduction of the four Yamanaka factors to cells cultured on feeder cells induces the development of colonies of cells with a variety of morphologies, but only a few of them have ES cell-like morphology and are thus identified as iPS cells. Usually, only those colonies with ES cell-like morphologies are picked up and further cultured for the generation of iPS cells, while colonies with a non-ES cell-like morphology are ignored because they are not considered to contribute to iPS cell generation and have thus not been analyzed in detail. Although these cells do not directly contribute to iPS cell generation, some intracellular changes might be caused by the introduction of the four Yamanaka factors, so that investigating the similarities and differences between these colonies and iPS cell colonies will be advantageous toward understanding iPS cells.

Analysis of the genes expressed by all colonies appearing during the generation of iPS cells was reported previously [6], but studies evaluating the morphologic characteristics in addition to the gene expression pattern of all the generated colonies have not been reported. Furthermore, ES cell-like colonies are most often judged under microscopic observation, and there are no objective criteria or parameters for identifying iPS cells. With regard to gene expression, the basis for iPS cell generation efficiency differs among reports; some reports calculate generation efficiency based only on alkaline phosphatase staining, whereas others are based on the expression of a reporter gene driven by the promoter of a single pluripotency marker such as Nanog or Oct3/4 [7], [8], [9]. Therefore, the reported generation efficiencies cannot be compared with each other and reliable markers or parameters for iPS cells must be defined.

In the present study, we transduced the four Yamanaka factors to adult human skin-derived fibroblasts using either four retroviral vectors encoding Oct3/4, Sox2, Klf4, and c-Myc, or a single polycistronic Oct3/4-Klf4-Sox2-c-Myc-GFP expressing viral vector, and subjected the fibroblasts to iPS cell generation procedures. After gene transduction, all generated colonies, including ES cell-like colonies, namely iPS cell colonies, were classified based on six morphologic parameters. We further performed gene expression analyses of each categorized colony and explored the elements available for identifying iPS cells. We show that the morphologic features specific for iPS cell colonies are objectively represented. Moreover, the expression of either Oct3/4 or Nanog, which are currently often used to identify iPS cells, is not appropriate for the identification of iPS cells, but rather the expression of endogenous Sox2 and Cdx2 is a reliable marker of iPS cells. Our results indicate that combined evaluation of morphologic parameters and gene expression of endogenous Sox2 and Cdx2 is useful for accurately identifying iPS cells and will greatly contribute to a better understanding of iPS cells.

## Results

Yamanaka four factors Oct3/4, Sox2, Klf4, and c-Myc were transduced to adult human dermal fibroblasts with four retroviral vectors or with a single polycistronic retroviral vector according to the original method [3], [10]. Five days after transduction, cells were transferred onto inactivated mouse embryonic fibroblasts and cultured in ES cell medium. Thirty days after the transduction with four retroviral vectors, 202 and 215 colonies were generated in two experiments respectively and each colony was picked up for further culturing. Ten and twelve colonies failed to be passaged, respectively, and the remaining 192 and 203 colonies were subjected to further analysis after two passages. In the case of the single polycistronic vector transduction, 199 and 192 colonies were obtained in two experiments respectively and all colonies were picked, passaged twice and analyzed.

We considered the morphologic characteristics that indicate ES cell-like cells and colonies, and set 6 parameters: 1) number of nucleoli, 2) nucleus to nucleolus (N/Nls) ratio 3) cell size, 4) cell density (cells/mm^2^), 5) nucleus to cytoplasm (N/C) ratio and 6) number of cell layers (multilayer or monolayer) of the colony, all of which are considered necessary elements for distinguishing ES cell-like colonies from among various other types of colonies. Following these parameters, we performed a morphometric analysis of randomly selected five cells from the central region of each colony.

Human ES cell colonies were firstly analyzed as a control, and resulted as follows: number of nucleoli 1.20±0.45; N/Nls ratio 2.20±0.17; cell size 43.70±3.61 µm^2^; cell density 6133.33±15.32 cells/mm^2^; N/C ratio 0.86±0.04; and single cell layer (Table 1).

**Table 1:** Classification of morphologic characteristics of colonies generated from human fibroblasts using four retroviral vectors encoding Oct3/4, Sox2, Klf4, and c-Myc.

	number of nucleolus	nucleus to nucleolus ratio	cell size	cell density/mm^2^	nucleus to cytoplasm ratio	number of layer	number of colonies obtained (Experiment 1)	number of colonies obtained (Experiment 2)
Col A	2.35±0.51	3.30±0.48	87.69±11.04	2166.67±29.21	0.59±0.07	multilayer	16	22
Col B	1.46±0.59	2.95±0.41	113.20±13.40	1863.06±22.49	0.53±0.06	multilayer	55	49
Col C	1.34±0.55	2.50±0.50	69.82±9.29	3048.96±26.14	0.60±0.07	multilayer	95	102
Col D	1.33±0.47	3.17±0.50	52.09±7.69	5091.67±21.65	0.6±0.05	multilayer	6	8
Col E	1.24±0.48	2.52±0.43	45.72±7.14	3493.33±24.03	0.59±0.07	multilayer	10	8
Col F	1.27±0.49	2.42±0.23	58.34±5.92	5266.67±41.01	0.64±0.06	multilayer	8	12
Col G (iPS cell)	1.20±0.45	2.19±0.17	43.50±2.96	5933.33±20.82	0.87±0.04	single	2	2
human ES cell	1.20±0.45	2.20±0.17	43.70±3.61	6133.33±15.32	0.86±0.04	single		

Each replicate represented 2×10^5^ transduced cells seeded onto a 60-mm dish containing feeder cells and cultured in Primate ES cell medium for thirty days.

**Figure 1:**
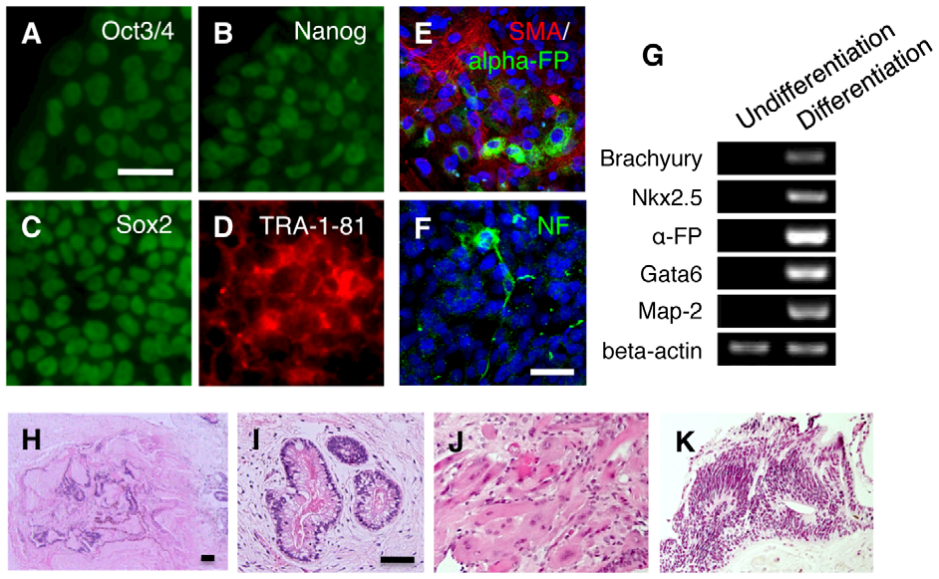
Characterization of iPS cell colony G. (A–D) Immunocytochemistry for (A) Oct3/4, (B) Nanog, (C) Sox2, (D) TRA-1-81 in iPS cell colony G. Scale bar = 100 µm. (E, F) EBs generated from colony G were plated on gelatin coated dishes containing DMEM/F12 medium supplemented with 20% knockout serum replacement. After 10 days, cell differentiation was confirmed by immunocytochemistry for mesodermal (smooth muscle actin; SMA) (E), endodermal (alpha-fetoprotein; alpha-FP) (E) and ectodermal markers (neurofilament; NF) (F). Scale bar = 100 µm. (G) RT-PCR of differentiation markers in undifferentiated iPS cell colony G (Undifferentiation) and embryoid bodies derived from iPS cell colony G. Differentiation). (H–K) Hematoxylin and eosin staining of teratoma formed by transplantation of iPS cell colony G into immunodeficient mice testis. (H), Low magnification of the formed teratoma (12 weeks after injection). Endodermal (I), mesodermal (J) and ectodermal (K) tissue were observed in the teratoma.

We classified an ES cell-like colony, namely an iPS cell colony, as colony G for both four retroviral vectors (2 colonies) and a single polycistronic vector (2 colonies). The mean parameter values of four colonies in colony G were: number of nucleoli 1.20±0.45; N/Nls ratio 2.19±0.17; cell size 43.50±2.96 µm^2^; cell density 5933.33±20.82 cells/mm^2^; N/C ratio 0.87±0.04; and single cell layer; indicating that each parameter in colony G was statistically identical to those of human ES cells (p<0.05 with Bonferroni's correction; Table 1). Cells in colony G expressed the pluripotency markers Oct3/4, Nanog, Sox2, and TRA-1-81 in immunocytochemistry (Fig. 1A–D), differentiated *in vitro* into cells representative of all three germ layers (Fig. 1E–G), and formed teratomas when transplanted into immunodeficient mouse testis (Fig. 1H–K).

**Figure 2:**
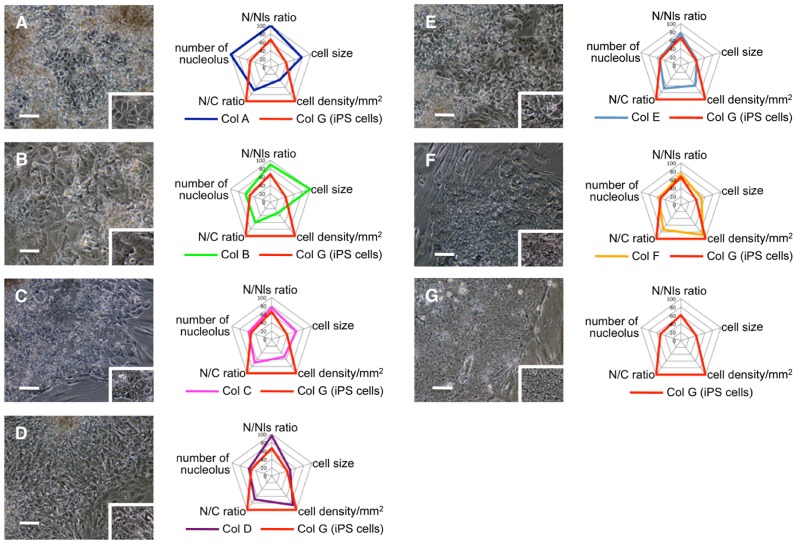
Morphometric analysis of colonies generated from human fibroblasts using four retroviral vectors encoding Oct3/4, Sox2, Klf4, and c-Myc. (A–G) Photographs and parameters of colonies A∼G. Graphs shows parameters of each classified colony, including that of iPS cell colony G. The numerical value in the graph indicate the ratio to the maximum value (setting 100 for maximum value) in each parameters. N/Nls = nucleus-to-nucleolus ratio; N/C = nucleus-to-cytoplasm ratio. Scale bars = 100 µm.

Besides two colonies that were already determined as colony G, remaining 190 and 201 colonies generated by four retroviral vectors were analyzed. The average and standard deviation of number of nucleolus, N/Nls ratio, cell size, cell density and N/C ratio in each colony were compared to those of colony G by t-test and all the colonies were sorted according to the number and content of parameters that showed significant statistical differences. As a result, colonies were categorized into 6 typical types, colony A∼F. When iPS cell colony G and the rest of the colonies A∼F were compared, iPS cells showed maximum value in cell density and N/C ratio, whereas cell size, number of nucleoli and N/Nls ratio were minimum. In addition to above 5 parameters, number of cell layer was also analyzed. In contrast to iPS cell colony G and ES cells that demonstrated single cell layer, colony A∼F piled up to form multiple layers. The parameters for each type of colony are shown in Table 1. The characteristics of each colony in terms of significant statistical differences to colony G are summarized as follows: colony A, number of nucleoli, N/Nls ratio, cell size, cell density, N/C ratio showed significant difference to those of colony G (p<0.01); colony B, N/Nls ratio, cell size, cell density, N/C ratio (p<0.01); colony C, cell size, cell density, N/C ratio (p<0.01); colony D, N/Nls ratio, cell density, N/C ratio (p<0.01); colony E, cell density, N/C ratio (p<0.01); colony F, cell size, N/C ratio (p<0.01) (Fig. 2A–G).

Gene expression of pluripotency markers was evaluated in colonies A∼G using reverse transcription-polymerase chain reaction (RT-PCR). We analyzed 10 genes that are reportedly expressed in both human ES and iPS cells [10], [11], [12], [13], [14]: endogenous Oct3/4, endogenous Sox2, Nanog, endogenous Klf4, endogenous c-Myc, FoxD3 (Forkhead box D3), Rex1 (Zfp42), Dnmt3b (DNA (cytosine-5-)-methyltransferase 3 beta), Abcg2 (ATP-binding cassette sub-family G member 2), and Cdx2 (caudal-type homeobox protein 2).

**Figure 3:**
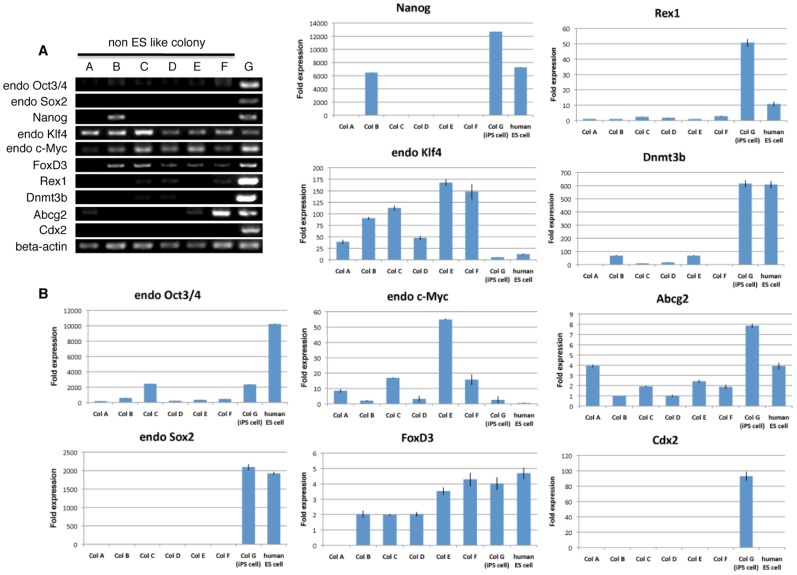
RT-PCR and Q-PCR of typical examples in each of colonies A∼F, iPS cell colony G and human ES cells. (A) RT-PCR analysis examined the expression of endogenous Oct3/4, Sox2, Nanog, Klf4, c-Myc, as well as FoxD3, Rex1, Dnmt3b, Abcg2 and Cdx2. Beta-actin was used as an internal control. (B) Q-PCR data for expression of endogenous Oct3/4, Sox2, Nanog, Klf4, c-Myc, as well as FoxD3, Rex1, Dnmt3b, Abcg2 and Cdx2. Beta-actin was used as an internal control.

Adult human dermal fibroblasts endogenously expressed Klf4 and c-Myc but not any other genes (data not shown). Colonies A∼F and iPS cell colony G also expressed Klf4 and c-Myc, indicating that these two genes were expressed from the beginning of the human dermal fibroblasts and their expression was maintained even after receiving the four Yamanaka factors, regardless of the colony type (Fig. 3A). Genes such as endogenous Oct3/4, endogenous Sox2, and Nanog, which cooperatively maintain self-renewal and pluripotent states in pluripotent stem cells, are known to be downregulated upon their differentiation [15], [16], [17]. In colonies A∼F, lower expression of endogenous Oct3/4 compared to iPS cell colony G was seen (Fig. 3A). It is noteworthy that, in colony B, Nanog expression was detected whereas endogenous Sox2 was not. Only iPS cell colony G expressed three sets of genes, namely endogenous Oct3/4, endogenous Sox2, and Nanog, and colonies A∼F consistently did not express Sox2 (Fig. 3A). As for FoxD3, Rex1, Dnmt3b, Abcg2, and Cdx2, none of the colonies A∼F expressed all 5 of these genes and in most cases, some were positive but others were negative. Importantly, like with Sox2, none of these colonies expressed Cdx2. Only iPS cell colony G expressed all the genes, including Sox2 and Cdx2 (Fig. 3A). These results were confirmed by quantitative-PCR (Q-PCR). In each fold expression calculation, the cyclethreshold (Ct) value of each genes expression in all colonies and human ES cells were compared to the average Ct value of each genes expression in adult human dermal fibroblasts (Fib.3B). Cdx2 is known to be required for differentiation of trophectoderm, and is reported not to be detected in undifferentiated human ES cells [18]. Consistent with this previous report, our result showed that the expression of Cdx2 was not detectable in undifferentiated human ES cells (Fig. 3B). On the other hand, the expression of endogenous Cdx2 was consistently detectable only in iPS cell colony G (Fig. 3B). Overall, the data obtained in Q-PCR showed same tendency to those in RT-PCR (Fig. 3B).

The formation of embryoid bodies (EBs) *in vitro* is a very important step for pluripotent stem cells to elicit their differentiation. EBs are usually formed in human pluripotent stem cells by culturing a single colony-derived small fragment containing 50 to 200 cells in suspension (Harvard Stem Cell Institute iPS Cell Core Facility; http://www.hsci.harvard.edu/ipscore/node/8). After culturing in suspension for 10 days, human pluripotent stem cells gradually expand and form EBs of at least 300 µm in diameter [19]. We tried to form EBs from colonies A∼G to verify their ability to differentiate. Large cell clusters with diameters greater than 300 µm were formed in iPS colony G (Fig. S1A), while cell clusters whose sizes were less than 90 µm were formed by single colony-derived small fragments in colonies A∼F, and these cell clusters did not continue to grow in suspension culture (Fig. S1B). When the formed cell clusters were transferred to gelatin-coated dishes, EBs from iPS cell colony G adhered to the bottom of the dish and the cells differentiated into endodermal (alpha-fetoprotein, GATA6), ectodermal (neurofilament, MAP-2), and mesodermal cells (smooth muscle actin, Brachyury, Nkx2.5) (Fig. 1E, F, G). On the other hand, all the cell clusters formed from colonies A∼F dispersed or degenerated so that adhesion of these cell clusters to the dish and their triploblastic differentiation were never confirmed. Based on these results, we concluded that the cell clusters that formed from iPS cell colony G were EBs because they expanded and differentiated into cells representative of all three germ layers, while cell clusters formed from colonies A∼F did not appear to form EBs, because they showed no further growth in suspension. Therefore, colonies A∼F were not considered to be iPS cells.

**Figure 4:**
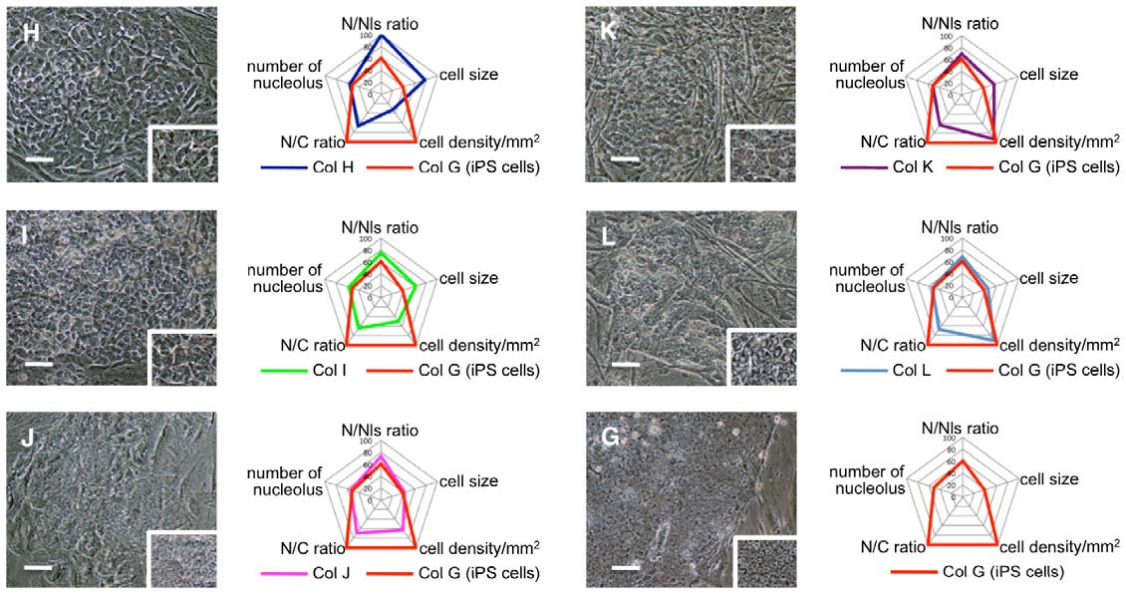
Morphometric analysis in colonies generated from human fibroblasts by using a single polycistronic Oct3/4-Klf4-Sox2-c-Myc-GFP expressing viral vector. (H–L, G) Photograph and parameters of colonies H∼L and G. Graphs shows parameters of each classified colony, including that of iPS cell colony G. The numerical value in the graph indicate the ratio to the maximum value (setting 100 for maximum value) in each parameter. Scale bars = 100 µm.

Because the transduction of all four individual Oct3/4, Sox2, Klf-4, and c-Myc retroviruses into the cells by the four retroviral vectors might not be successful, we repeated the analysis by transducing a single polycistronic viral vector encoding Oct3/4-Klf4-Sox2-c-Myc-GFP into the cells [10]. Transduced cells were isolated by fluorescence-activated cell sorting based on green fluorescent protein gene expression, and then subjected to the iPS cell generation procedure. Cells in 199 and 192 colonies were morphometrically analyzed based on the above six parameters. The morphometric data obtained from all of these colonies, except for the colonies categorized as colony G, were statitstically different from those of the colonies generated by four retroviral vectors encoding Oct3/4, Sox2, Klf4, and c-Myc individually, namely colonies A∼F. and therefore, we newly categorized them into five subtypes, that is, colonies H∼L. Colony H showed statistically significant difference to colony G in N/Nls ratio, cell size, cell density and N/C ratio (p<0.01). In colony I, cell size, cell density, N/C ratio (p<0.01), in colony J, cell density and N/C ratio (p<0.01), colony K, cell size and N/C ratio (p<0.01), and colony L, only N/C ratio (p<0.01) (Table 2, Fig. 4H–L). As for the number of cell layers, colonies H, I and K had a monolayer while colonies J and L had multiple cell layers (Table. 2).

**Table 2:** Classification of morphologic characteristics in colonies generated from human fibroblasts using a single polycistronic viral vector.

	number of nucleolus	nucleus to nucleolus ratio	cell size	cell density/mm^2^	nucleus to cytoplasm ratio	number of layer	number of colonies obtained (Experiment 1)	number of colonies obtained (Experiment 2)
Col H	1.30±0.50	3.60±0.60	87.50±9.36	1933.34±19.05	0.58±0.06	single	16	11
Col I	1.33±0.54	2.71±0.46	70.01±7.13	2962.96±20.91	0.56±0.05	single	64	66
Col J	1.26±0.47	2.65±0.45	47.09±7.34	3745.00±24.86	0.61±0.07	multilayer	71	69
Col K	1.20±0.45	2.50±0.49	65.06±5.69	5466.67±40.92	0.55±0.05	single	16	23
Col L	1.25±0.47	2.48±0.39	51.55±5.91	5383.84±34.67	0.59±0.06	multilayer	30	21
Col G (iPS cell)	1.20±0.45	2.19±0.17	43.50±2.96	5933.33±20.82	0.87±0.04	single	2	2

Each replicate represented 2×10^5^ GFP positive cells seeded onto a 60-mm dish containing feeder cells and cultured in Primate ES cell medium for thirty days.

**Figure 5:**
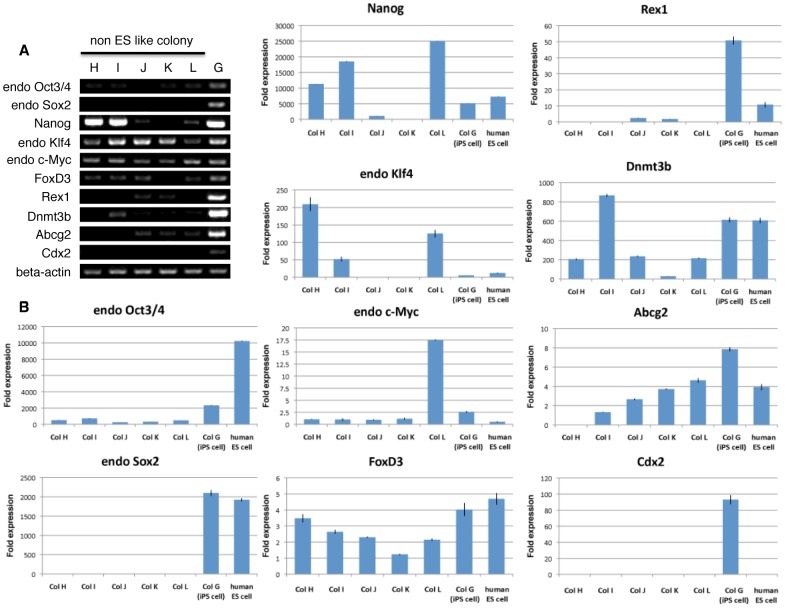
RT-PCR and Q-PCR of typical examples in each colonies H∼L and iPS colony G and human ES cells. (A) RT-PCR analysis examined the expression of endogenous Oct3/4, Sox2, Nanog, Klf4, c-Myc, as well as FoxD3, Rex1, Dnmt3b, Abcg2 and Cdx2. Beta-actin was used as an internal control. (B) Q-PCR data for expression of endogenous Oct3/4, endogenous Sox2, Nanog, endogenous Klf4, endogenous c-Myc, FoxD3, Rex1, Dnmt3b, Abcg2 and Cdx2.

RT-PCR demonstrated that colonies H, I, K, and L expressed endogenous Oct3/4 with weaker intensity than that in iPS cell colony G, and that colonies H, I, J, and L expressed Nanog while none of colonies H∼L expressed endogenous Sox2 (Fig. 5A). These findings were further confirmed by Q-PCR. The expression of endogenous Oct3/4 was detected in all colonies. The expression level of endogenous Oct3/4 was three to ten times lower than that in iPS cell colony G, and that of Nanog in colonies H, I and L ranged from being almost equivalent to up to two to five times higher than that in iPS cell colony G (Fig. 5B). However, the expression of Sox2 was consistently undetectable in colonies H∼L (Fig. 5B).

Colony J expressed FoxD3, Rex1, Dnmt3b, and Abcg2, while colonies H, I, K and L expressed some combination of two or three of these genes (Fig. 5A). Importantly, like Sox2, Cdx2 expression could not be detected in colonies H∼L, while only iPS cell colony G expressed Cdx2 (Fig. 5A,B). Furthermore, we performed suspension culture in colonies H∼L. As described above, single colony-derived small fragments from colonies H∼L did not show further growth to form EB-like cell clusters in suspension culture (data not shown).

## Discussion

The polycistronic vector reduced the variation in morphologic categorization but induced different morphologic features and gene expression patterns compared with those obtained with individual Oct3/4, Sox2, Klf-4, and c-Myc retroviruses. In our case, all the colonies arising during iPS cell generation could be categorized morphologically into 1 of 12 colony types. Common among colonies A∼L, the non-ES cell-like colonies did not express endogenous Sox2 and Cdx2, while in contrast, iPS cell colony G consistently expressed both genes.

We conclude that the criteria to determine iPS cell colonies can be summarized as follows: number of nucleoli 1.20±0.45; N/Nls ratio 2.19±0.17; cell size 43.50±2.96 µm^2^; cell density 5933.33±20.82 cells/mm^2^; N/C ratio 0.87±0.04; and single cell layer; and cells positive for endogenous Sox2 and Cdx2. For further confirmation, the expression of other pluripotency-related factors, endogenous Oct3/4, Nanog, FoxD3, Rex1, Dnmt3b, and Abcg2, will be useful.

Up to now, iPS cell colonies are visually identified as the flat cell colonies with defined edge and tightly packed cells [3]. Such identification would be dependent on ones subjective judgment and would not be reliable objective criteria. In this report, based on morphologically objective parameters, we newly demonstrate that iPS cell colony possess particular morphological character compared to non-iPS cell colonies in that iPS cells show maximum value in cell density and N/C ratio and minimum in cell size, number of nucleoli and N/Nls ratio. In addition, iPS cell colony can be specifically identified by their expression of Sox2 and Cdx2. Thus, both morphologic features and expression both of Cdx2 and Sox2 would improve the precision of iPS cell generation ratio. However, Sox2 is reported to be expressed in neural stem cells or other tissue stem cells [20], [21], [22]. Therefore, in the case of generating iPS cells from stem cells that already express endogenous Sox2, Cdx2 expression and morphologic parameters will be helpful for iPS cell identification. In addition, since Cdx2 is not expressed in human ES cells, this gene is considered specific for iPS cells. The function of Cdx2 in iPS cells need to be clarified in the future.

Previous studies reported gene expression analyses that were performed using live cell imaging and Q-PCR in both ES-like and non-ES like colonies [6], [23]. In these reports, Nanog, Oct3/4, and alkaline phosphatase were expressed in various types of colonies and considered to be inadequate markers to identify iPS cells [6], [23]. In this study, however, we showed that colonies other than iPS cells express both Nanog and endogenous Oct3/4, and therefore iPS cell identification based on Nanog or Oct3/4 is not reliable method. Furthermore, that may contaminate non-iPS cells into iPS cell count, leading to the overestimation of iPS cell generation ratio.

Up to now, iPS cell generation efficiency was often calculated based on the expression of reporter genes such as GFP under the control of certain gene promoters [8], [9]. However, none of the studies examined whether the expression of these reporter genes are truly appropriate to identify iPS cells. At least in the case of Nanog and Oct3/4, our result suggested that the expression of these reporter genes is not reliable for identification of iPS cells. Furthermore, it is reported that the expression of reporter gene under the control of specific gene promoter do not reflect the true expression of the specific gene in some cases [24], [25]. In this sense, whether the reporter gene under the control of Cdx2 appropriately function for iPS cell generation needs to be clarified in the future.

Objective criteria for identifying iPS cells have not yet been established. Selecting iPS cells or calculating the iPS cell generation ratio using accurate and objective method is necessary for standardization of iPS cell research, and, from this viewpoint our results will contribute to the establishment of unified criteria for objectively identifying iPS cells.

## Materials and Methods

### Ethics Statement

All animal experiments were approved by the Animal Care and Experimentation Committee of Tohoku University Graduate School of Medicine. The entire study was conducted in accordance with the Declaration of Helsinki. Human ES cell line Kyoto hESC-1 was obtained from the Institute for Frontier Medical Science of Kyoto University (Japan) with approval for hESC use granted by the Ministry of Education, Culture, Sports, Science, and Technology of Japan.

### Gene introduction and iPS cell generation

Normal human dermal fibroblasts were obtained from Lonza (Basel, Switzerland). Cells were cultured in alpha-minimum Essential Medium Eagle Modification (Sigma, St. Louis, MO) containing 10% fetal bovine serum and 0.1 mg/ml kanamycin at 37°C and 5% CO_2_. Induced pluripotent stem cells were generated as reported by Takahashi et al [3]. The open reading frames of human Oct3/4, Sox2, Klf4, and c-Myc were amplified by RT-PCR using PrimeSTAR HS DNA Polymerase (TaKaRa, Shiga, Japan) and inserted into pMXs retroviral vectors. These plasmids were transfected into PLAT-A retroviral packaging cells followed by incubation in Dulbecco's Modified Eagle Medium containing 10% fetal bovine serum. Forty-eight hours after transfection, the viral supernatant was collected and filtered through a 0.45-µm filter. The virus-containing flow-through was concentrated by centrifugation at 8000× g for 16 h at 4°C.

For iPS cell generation, human fibroblasts were seeded at 2×10^5^ cells in a 60-mm dish. The next day, concentrated virus plus 4 µg/ml Polybrene (Nacalai Tesque, Kyoto, Japan) was added to the medium. Twenty-four hours after transduction, the virus-containing medium was replaced with new medium without virus. After 5 days, fibroblasts were trypsinized and 1×10^5^ cells were transferred onto inactivated mouse embryonic fibroblasts cultured in a 100-mm diameter dish. The next day, the medium was replaced with Primate ES cell medium (ReproCELL, Kanagawa, Japan) supplemented with 4 ng/ml basic fibroblast growth factor (Peprotech, Rock Hill, NJ). The medium was changed every other day. Thirty-five days after transduction, all colonies were picked and individually transferred onto inactivated mouse embryonic fibroblasts in 4-well plates. Detailed information for the single polycistronic retroviral vector encoding the four transcription factors was described in a previous report [10].

### Statistical analysis

Morphometric data of randomly selected five cells from the central region of each generated colony are analyzed and presented as the mean ± s.d. Cell density and cell size were analyzed by t-test with Bonferroni's correction for multiple comparisons. Number of nucleoli and the N/C and N/Nls ratios were analyzed using the Chi-square test and Mann-Whitney U test, respectively, both with Bonferroni's correction. In the Bonferroni's correction, the actual level of significance was corrected to and accepted as p<0.01 to correct for the five pairwise comparisons.

### RNA extraction and RT-PCR

Three individual colonies were randomly selected from each colony subtype and subjected to RT-PCR. Total RNA was purified with an RNeasy Mini Kit (QIAGEN, Hilden, Germany). RNA (500 ng) was reverse-transcribed using a SuperScript VILO cDNA Synthesis Kit (Invitrogen, Carlsbad, CA) according to the manufacturer's instructions. The PCR reactions were performed using Ex Taq DNA Polymerase (TaKaRa). The primer set for a pluripotency marker FoxD3 was:

sense, 5′- CTGCCTCTCCCCAATTTCCT-3′ and antisense, 5′- TCCCATCCCCACGGTACTAA -3′.The RT-PCR primer sets for the other genes were described previously [10]. The primer sets for Oct3/4 and Sox2 were designed from the untranslated region, which does not detect the retroviral transcripts, so that the primers specifically detect the transcripts from the endogenous Oct3/4 and Sox2 genes.

The signal intensity for each factor occasionally differed among colonies, but the gene expression pattern was same among colonies that belong to the same subtype. Therefore, one colony was randomly selected from each colony subtype and demonstrated in figure 1H.

### Q-PCR

Three individual colonies were randomly selected from each colony subtype and subjected to Q-PCR. cDNA was synthesized using a High Capacity cDNA Reverse Transcription Kit (Applied Biosystems, Foster, CA). After cDNA preparation, genes of interest were amplified using TaqMan gene expression assays (Applied Biosystems). Q-PCR was performed with a 7300 real-time PCR system (Applied Biosystems) using the following PCR primer sets, Oct3/4 (Hs03005111_g1), Sox2 (Hs01053049_s1), Nanog (Hs02387400_g1), Klf4 (Hs00358836_m1), c-Myc (Hs99999003_m1), FoxD3 (Hs00255287_s1), Rex1 (Hs00399279_m1), Dnmt3b (Hs00171876_m1), Abcg2 (Hs01053790_m1), Cdx2 (Hs01078080_m1) and beta-actin (Hs99999903_m1). Data were processed using the ΔΔCt method.

### EBs formation from iPS cells and non-ES like colonies

iPS cells and non-ES like colonies were harvested by mechanical pick-up. The clumps of cells were transferred to poly 2-hydroxyethyl methacrylate-coated dish in Dulbecco's Modified Eagle Medium/F12 containing 20% knockout serum replacement (Invitrogen), 1×10^−4^ M nonessential amino acids, 1×10^−4^ M 2-mercaptoethanol, 2 mM L-glutamine, and 0.1 mg/ml kanamycin. The medium was changed every other day. After 10 days, EBs were transferred to gelatin-coated plates and cultured in the same medium for another 10 days.

### Teratoma formation

iPS cells were suspended at 1×10∧7 cells in 0.02 M PBS and were injected using glass microtubes into the testes of 8 weeks old NOG mice [NOD/Shi-scid [26] IL-2RγKO Jic mice; ICLAS Monitoring Center, Japan]. As negative control, testes were injected with mouse embryonic fibroblasts treated with mitomycin C. 12 weeks after injection, all mice were killed and fixed with 4% PFA in 0.1 M PBS. These fixed testes were embedded in paraffin, and cut into sections 10 µm thick. These sections were stained with hematoxylin and eosin.

## Supporting Information

Figure S1 *In vitro* embryoid body (EB) formation. (**A**) EBs generated in suspension culture from iPS cell colony G. (B) Cell clusters generated in suspension from colony C as representative of colonies A∼F. Scale bars = 100 µm.
(TIF)
