# Supplementary figures and images for: Morphologic and Gene Expression Criteria for Identifying Human Induced Pluripotent Stem Cells
Source: PLoS One. 2012 Dec 13;7(12):e48677. doi: 10.1371/journal.pone.0048677 (PMC3521736; doi:10.1371/journal.pone.0048677)

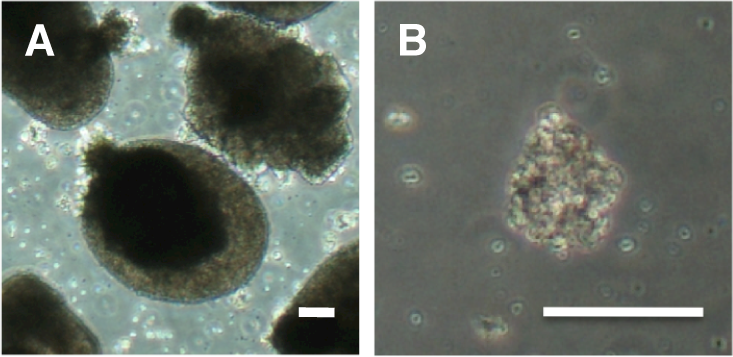

Supplement: Figure S1 — In vitro embryoid body (EB) formation. (A) EBs generated in suspension culture from iPS cell colony G. (B) Cell clusters generated in suspension from colony C as representative of colonies A∼F. Scale bars = 100 µm. (TIF) [file pone.0048677.s001.tif]
